# On Stricture of the Urethra and Rectum

**Published:** 1873-10-01

**Authors:** W. C. Lyman


### ON STRICTURE OF THE URETHRA AND RECTUM.

REPORT READ BEFORE THE CHICAGO SOCIETY OF PHYSICIANS AND SURGEONS, AUG. 25, 1873, BY W. C. LYMAN, M.D.

In making some statements relative to stricture of the urethra, I will avoid anything concerning diagnosis, cause, locality, or pathological anatomy, and confine myself solely to the methods of treatment, prefacing with the remark that established methods sometimes give place to new ones, apparently promising, but which do not stand the test of experience, when a return to former practice occurs. I refer especially to those cases requiring either gradual or immediate dilatation, and not to those that require operation by incision, as they may be considered to constitute an entirely different class. I can recall about 150 cases of this form of disease, and about one-third were treated by immediate dilatation. These are sufficient in number to enable one to arrive at a conclusion reasonably correct as to the utility of the two methods. I say, unhesitatingly, that I prefer the gradual method for the following reasons:

*First*—The gradual method, carefully put in practice, does not keep the patient from his ordinary business.

*Second*—The sure requirement of anæsthesia does not alarm the patient.

*Third*—There is complete absence of danger from shock, which attaches itself to all cases of the immediate operation, particularly in persons advanced to middle life.

*Fourth*—The complete cure by the gradual method is, at least, as expeditious as the other, there being frequently a chronic discharge which lasts a long time.

*Fifth*—The greater liability to return of the stricture, by contraction of the cicatrix made by rupture, and by the continual presence of induration, which formerly constituted the stricture, rendering a repetition of the operation frequently necessary.

*Sixth*—Immediate dilatation is far more painful, both during and after the operation, than the other plan.

*Seventh*—There is loss of symmetry by rupture, that is wholly avoided by the gradual absorbtion of the thickening of the urethral mucous and submucous tissues by equable pressure.

The immediate result of the rupture of a stricture is very flattering. The urine which was voided in a small and irregular stream, through a passage admitting only a No. 4 sound, perhaps, passes, at first, in a large, full stream. This will continue but a few days, unless a full-sized sound is used daily. The passage lately so widely dilated rapidly contracts if left alone ; and the use of the sound is, almost without exception, excessively painful, and often excessively difficult, from the ruptured and unsymmetrical condition of the urethra. These conditions, which so nearly always obtain, contrast very unfavorably with the easy and almost entirely painless journey to health by the other path. I wish, furthermore, to state that a great many cases of what are really inflammatory or true strictures can be cured without any effort at dilatation whatever. I refer to simple inflammatory thickening of the urethral lining, a sequel of a gonorrhœa. This produces a decided diminution of the urethral calibre, and is a forming stricture. These cases will yield to the same methods that are successfully used in chronic inflammations of other similar tissues, as the conjunctival, pharyngeal, or nasal lining membranes.

It has long been the opinion of the writer that the ordinary means in use by the profession for treatment of stricture of the rectum are far inferior in utility to those which cases of this formidable difficulty really demand. We find the rectum, from one and one-half to three inches from the anus, narrowed, thickened, and indurated, till its elasticity, calibre and contractibility are almost, if not entirely, lost, and the patient finds life to be one long period of anxiety and distress.

Dilatation with the bougie, although it can do much, must stop far short of anything like a complete cure. The greater part of the thickening remains, and the disposition to a return of the symptoms is exceedingly strong, as the largest bougie that can pass the sphincter can never fully restore the calibre or the shape of the rectal space.

The use of the bi-valve dilator, shaped like a long speculum, can accomplish more; but *its* power is limited; and if there are internal hæmorrhoids, or varix of the hæmorrhoidal veins, dangerous hæmorrhage may occur, and, in fact, has occurred. It can readily be seen how this may occur with the force operating only in opposite directions, and not radiating from the center. Incisions of the fibrous bands of the stricture have been practiced, followed by the use of the bougie, as in ordinary cases, and with the same result. If this partial dilatation and partial absorption of plastic deposit can be obtained, it is fair to conclude that if the efforts could be continued beyond their usual limits, and the rectum be dilated to its full capacity, persistence in the treatment would be rewarded by the return of the rectal walls to their natural degree of thickness and elasticity. This has been lately more fully accomplished than ever before, by the following means: Five or six rubber "capotes" or "condoms" are drawn one over the other, and a director, such as a small piece of whalebone, protected at the end by a piece of sponge, and long enough to pass up through the stricture, is left within the elastic sac thus made, the open end of the instrument being securely fastened upon a small metal or ivory tube, that can be connected with an ordinary Davidson's syringe. The elastic sac already passed through the stricture is filled with water by the syringe, and the necessary degree of dilatation accomplished by successive efforts.

The introduction of the empty sac is easy enough, unless the constriction is unusually tortuous.

The evident advantages of this method are:

An entire absence of danger from hæmorrhage, as the sac fills all parts, and, by mechanical pressure, prevents it.

The very slight degree of pain inflicted, and the consequent absence of fear on the part of the patient.

The production of an equally powerful expansive force in every direction, acting symmetrically, producing true dilatation more than rupture of tissue.

The possibility of continuing its use till the rectum is restored to its normal calibre.

The impossibility of inflicting any injury upon neighboring organs or tissues, or producing undue dilatation or loss of power of the sphincter muscle.

The single case that it has been my fortune to treat in the manner above stated, occurred in a young married woman about thirty years of age, who has suffered for the past ten years with some degree of this disability. When first seen, the opening through the mass constituting the stricture was too small to admit more than a No. 24 sound, of the French scale. This was gradually dilated, first by the bougie, and then by a bi-valve dilator, till the blades of the latter instrument could be easily opened one and one-half inches. Dangerous hæmorrhage then occurred, following, immediately, an effort at dilatation, and greatly alarmed the patient, who ceased making any further effort with a view to her recovery. She went on well for a period of two years, with greatly improved general health, but the gradual contraction necessitated another resort to dilatation; and I am glad to be able to report that the new method has greatly and expeditiously relieved the patient, and that it is fair to conclude that persistence in the same means will finally quite overcome the difficulty.

